# Electrophysiological responses of the clam (*Ruditapes decussatus*) osphradium to amino acids and alarm cues

**DOI:** 10.1007/s00359-025-01757-2

**Published:** 2025-09-05

**Authors:** Ana Rato, Joana Costa, Diana Gonçalves, Domitília Matias, Sandra Joaquim, Peter C. Hubbard

**Affiliations:** 1 Centre of Marine Sciences (CCMAR), University of Algarve, Campus de Gambelas, Faro, 8005- 139 Portugal; 2 Department of Sea and Marine Resources, Portuguese Institute for Sea and Atmosphere (IPMA, I.P.), Av. 5 de Outubro s/n, Olhão, 8700-305 Portugal; 3 University of Algarve, Campus de Gambelas, Faro, 8005-139 Portugal; 4 Interdisciplinary Centre of Marine Environmental Research (CIIMAR), University of Porto, Terminal de Cruzeiros do Porto de Leixões, Av. General Norton de Matos s/n, Matosinhos, 4450-208 Portugal

**Keywords:** Electrophysiology, Bivalve, Amino acids, Alarm cues, Predator-released kairomones, Osphradium

## Abstract

Chemical sensing of the surrounding environment is crucial for many aspects of bivalve biology, such as food detection and predator avoidance. Aquatic organisms strongly depend on chemosensory systems; however, little is known about chemosensory systems in bivalves. To understand how the carpet shell clam (*Ruditapes decussatus*) senses its surrounding chemical environment, we used an electrophysiological technique – the electro-osphradiogram – to assess the sensitivity of the osphradium to different putative odorants (amino acids, bile acids) and odours (predator-released cues and signals from con- and heterospecific bivalves). The clam osphradium was sensitive to most proteinogenic L-amino acids, evoking negative, tonic, and concentration-dependent responses. However, acidic amino acids (L-glutamic and L-aspartic acid), L-arginine and bile acids (cholic, taurocholic and taurolithocholic acid) failed to evoke any response. Surprisingly, while cues from injured bivalves (con- and heterospecific) evoked strong responses, predator-released cues (green crab, *Carcinus maenas*) failed to elicit any response, whether fed or unfed. That predator-released cues failed to evoke an electrophysiological response in the clam osphradium may indicate that they use cues released by injured prey – alarm cues – to avoid predation and/or that predators are detected by different sensory modalities. Indeed, the behavioural assays, performed to understand how clams make use of such sensory inputs, revealed that the activity index decreased after exposure to water conditioned with injured conspecifics, suggesting the origin of such alarm cues. Further research is needed to identify the chemical nature of these cues. We suggest that the electro-osphradiogram will be a useful tool in this endeavour.

**Supplementary Information:**

The online version contains supplementary material available at 10.1007/s00359-025-01757-2.

## Introduction

Sensing chemical cues in the environment is essential for several aspects of animal life, such as food detection, predator avoidance and finding conspecifics for reproduction (Derby and Sorensen 2008; Hay 2009). Hence, sensory systems represent a crucial interaction between animals and their environment (Lindberg and Sigwart 2015). Most organisms have evolved chemosensory systems to detect chemical cues involved in these processes (Ache and Young 2005). While terrestrial organisms almost exclusively detect volatile airborne and water–soluble chemicals (Ache and Young 2005), chemoreception in aquatic organisms is open to water-soluble chemicals in general (Ache and Young 2005; Mollo et al. 2017). This means that aquatic animals may detect different types of odorants from terrestrial animals.

Due to the physical characteristics of the marine environment, e.g., high turbidity and low light levels, aquatic organisms are heavily dependent on these chemosensory systems. Although the chemosensory systems are well characterized in fishes and crustaceans, (the olfactory system and the antennules, respectively), little is known about chemosensory systems in bivalves. In bivalves, as in gastropods, chemoreception has been suggested to be mediated by the osphradium (Haszprunar 1987; Nezlin and Voronezhskaya 1997; Lindberg and Sigwart 2015; Rato et al. 2023). The osphradium is located in the gill axis (Beninger et al. 1995), typically innervated from the visceral ganglion by the ctenidial nerve. It is composed of a sensory epithelium and ganglion connected to the central nervous system by the osphradial nerve (Nezlin and Voronezhskaya 1997; Lindberg and Sigwart 2015).

Some metabolites (e.g., sugars, proteins and amino acids) indicate the presence of food (Hay 2009). Amino acids are potent odorants for many aquatic animals, involved in food location and identification, and trigger feeding behaviour (Hara 1994; Velez et al. 2007); several aquatic taxa: fishes (Hara 2006), amphibians (Arzt et al. 1986), crustaceans (Fuzessery and Childress 1975; Kamio and Derby 2017) and molluscs (e.g. Bailey and Laverack 1966; Croll 1983; Rato et al. 2023) have strong sensitivity to amino acids (Cozzolino et al. 2024). Therefore, the current study used a range of amino acids to validate the method for electrophysiological recording from the osphradium.

In bivalves, as in other aquatic animals, chemical signals indicative of predation are released by injured conspecifics or closely-related heterospecifics, or are released by the predators themselves (Griffiths and Richardson 2006; Smee and Weissburg 2006a; Scherer et al. 2016). Alarm cues – released by damaged or disturbed conspecifics under predation - are responsible for triggering behavioural and/or morphological responses, such as thickening of the shell (Fässler and Kaiser 2008), so that predation risk is reduced (Wisenden 2000). These cues provide reliable and precise information of an imminent threat (Scherer et al. 2016). Chemical cues released by the predators themselves that alert potential prey to their presence – kairomones - may also evoke antipredator behaviour (Ferrari et al. 2010). This may depend on the identity of the predator detected (Castorani and Hovel 2016). However, the chemicals involved in these processes have yet to be identified.

Several behavioural strategies are used by bivalves to avoid predation, including aggregation (Nicastro et al. 2007), decrease of feeding activity (e.g. Smee and Weissburg 2006a), valves closure (Dzierżyńska-Białończyk et al. 2019), and burying more deeply in the substrate (Flynn and Smee 2010). The hard-shell clam (*Mercenaria mercenaria*) reduces feeding in response to cues from injured conspecifics or water conditioned by recently fed blue crabs (*Calinectes sapidus*), regardless of their diet (Smee and Weissburg 2006a). Similarly, to escape from predators, soft-shell clams *Mya arenaria* burrow deeper into the sediment (Flynn and Smee 2010). Nevertheless, predator avoidance incurs costs as it requires prey to allocate resources to defence or avoidance behaviours, therefore compromising growth, feeding and reproduction (Schoeppner and Relyea 2005; Hay 2009; Johnson and Smee 2012; Scherer et al. 2016).

As prey often use chemical cues to evaluate predation risk (predator or prey-released cues or a combination of both), prey need to be able to distinguish between cues and evaluate the associated risk level and respond appropriately (Johnson and Smee 2012). The current study, therefore, aimed to understand how the carpet shell clam (*Ruditapes decussatus*) – a species of high economic value – senses the surrounding chemical environment by exposing them to different odorants (amino acids and bile acids) and odours (predator-released kairomones and putative alarm cues from conspecific and heterospecific bivalves) and recording of the electro-osphradiogram (EOsG). Based on these recordings, a set of behavioural assays was conducted to evaluate how clams make use of such sensory input.

## Materials and methods

Adult carpet shell clams (*Ruditapes decussatus*) (10.84 ± 5.86 g total weight and 3.60 ± 0.55 cm length), hereafter ‘clams’, were collected from the Ria Formosa lagoon (southern Portugal). Immediately prior to electro-osphradiogram (EOsG) recordings, the right valve was carefully removed. Ethical approval is not required in Portugal for work on non-cephalopod molluscs.

### Preparation of stimuli: amino acids, serotonin and bile acids

All twenty proteinogenic L-amino acids were tested. Each was prepared at an initial concentration of 10^− 3^ M, directly dissolved in charcoal-filtered natural seawater. Charcoal-filtered natural seawater was used as blank (negative control), while L-cysteine (10^− 3^ M) was used as standard (positive control) since it evoked consistent and strong responses in preliminary experiments.

The neurotransmitter serotonin (5-HT, 10^− 3^ M) – an inducer of spawning in bivalves (Gibbons and Castagna 1984) – and bile acids [cholic acid, taurocholic acid (TCA), and taurolithocholic acid (TLC)] – potent odorants for fish (Buchinger et al. 2014) – were also used as stimuli. Bile acids were first dissolved in methanol (10^− 2^ M) and diluted (1:1000) in charcoal-filtered natural seawater to a final concentration of 10^− 5^ M. In this case, a diluted solution of methanol (1:1000) was used as blank.

### Predator-released cues and signals from conspecific and heterospecific bivalves

In preliminary experiments, clams were exposed to crab-conditioned water (*Carcinus maenas*) and water wherein crabs were fed with clams or oysters. These experiments showed that crab-conditioned water failed to evoke a response in the osphradium, whereas water conditioned by actively feeding crabs elicited a strong response. Therefore, to understand the origin of such cues (predators and/or injured prey), two different approaches were used.

Firstly, crabs unfed for 48 h were divided into two groups (*N* = 6 per group) and transferred to an aquarium with filtered natural seawater (1 L per crab). One group (15.93 ± 4.00 g mean ± SD) was fed with mussel flesh, in a ratio of 3% of crab weight (McGaw and Penney 2014) (0.48 ± 0.08 g per crab), while the other group (13.38 ± 4.24 g mean ± SD) remained unfed. After an initial 1-hour conditioning period, crabs were briefly washed with clean seawater and transferred to a second aquarium with clean natural seawater, where they were kept for an additional hour. Crab-conditioned water was then collected from this second aquarium.

The second approach used water conditioned with conspecific and heterospecific bivalves as stimuli. Adult clams (*Ruditapes decussatus*), oysters (*Magallana gigas*) and mussels (*Mytilus galloprovinciallis*) were used to condition seawater for 1 h (1 g.10 mL^− 1^) in three different scenarios: (1) live bivalves (with closed valves), (2) live bivalves with one valve removed but otherwise undamaged, and (3) dead bivalves with chopped flesh.

### Electro-osphradiogram (EOsG) recording

**Fig. 1:**
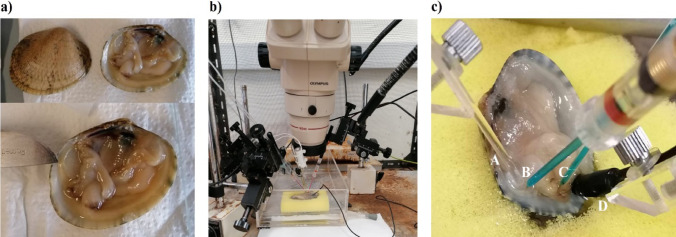
**a** Position of the osphradium (the scalpel indicates the approximate location). **b** Electro-osphradiogram apparatus. **c** Placement of the electrodes and stimulus-delivery tube; (A) stimulus-delivery tube; (B) recording electrode; (C) reference electrode; and (D) silver/silver chloride pellet for earth connection

The chemosensory activity of the osphradium was recorded by the electro-osphradiogram (EOsG), developed to record from the osphradium in bivalves as previously described by Rato et al. (2023). Firstly, one valve was removed by careful cutting of adductor muscles. During EOsG recording, clams were kept in an experimental chamber (Fig. 1) with continuous irrigation of the osphradium by clean seawater under gravity, *via* a glass tube, at a rate of 10 mL.min ^− 1^. Stimuli were introduced into this flow *via* a remotely operated solenoid valve (for six seconds). The recording electrode (Fig. 1) was placed close to the posterior adductor muscle, usually between the two siphons, and the reference electrode was placed nearby on the mantle. The optimal positions for electrodes and stimulus-delivery tube were determined using 10^− 3^ M L-cysteine as stimulus, placing the recording electrode where the response was largest. The electrodes, made from borosilicate glass micropipettes, were filled with 3 M NaCl in 4% agar and connected to the DC amplifier via Ag/AgCl pellets in 3 M KCl. Clams were connected to earth *via* a silver/silver chloride pellet placed in the mantle cavity.

The D.C. signal was amplified (x 2000 or x 5000) using a Neurolog head-stage NL100 and NL109 amplifier, with the low-pass filter (NL 125) set at 30 Hz (https://www.digitimer.com/). The signal was then digitized (Digidata 1440 A, Molecular Devices, Sunnyvale, California, USA; https://www.moleculardevices.com/) and recorded on a PC running AxoScope ^TM^ software (version 12.1, Molecular Devices). Stimuli were given in a varied order but in order of increasing concentration (10^− 6^ M to 10^− 3^ M). A minimum of 1 min was allowed between successive stimuli. Blank and standard solution (10^− 3^ M L-cysteine) responses were recorded at regular intervals throughout the recording period. Experiments took place in a Faraday cage.

### Behavioural assays

In each individual assay, five clams were placed in an aquarium (14.5 cm x 14.5 cm) with charcoal-filtered natural seawater (salinity 35, pH 8.20 ± 0.01) at room temperature, with aeration, fed 50 mL of a microalgae mix and left overnight. Before starting behavioural observations, faeces were gently removed from the aquarium. To exclude any potential disturbance in clam behaviour due to cleaning, behavioural trials started 30 min later.

Five behavioural traits (Table 1; fig. S1) were observed for 150 min and scored as 1 or 0 (presence or absence, respectively). For example, if a clam had its valves open it would be scored 1, if closed 0. These observations were made before adding the stimuli (T0) and every 10 min thereafter. Stimuli were added at T0, T60 and T120. Filtered seawater was used as negative control (‘Control’; *N* = 9), while microalgae mix (*Skeletonema costatum*,* Tisochrysis lutea* and *Chaetoceros calcitrans*; 1:1:1) was used as positive control (‘Microalgae mix’; *N* = 14). Water conditioned with (1) live conspecifics with one valve removed – ‘Live and opened’ (*N* = 9) – and (2) dead conspecifics with chopped flesh – ‘Dead and chopped’ (*N* = 9) – (prepared as aforementioned) were used as stimuli, in a dilution of 1:100. Given the experimental constraints, it was not possible to do the observations blind.

**Table 1:** **–** Description of the behavioural traits observed during the trial

Behavioural trait	Description
Valve	Observation of the valve open.
Mantle	Observation of the open valve, where it was possible to see the mantle. It occurs concomitantly with the open valve.
Siphon activity	Observation of the siphons; siphons extended.
Foot	Observation of the extended foot.
Filtration	Observation of siphons extended where it was possible to observe filtration through the opening of the inhalant siphon and through water movement.

The frequency of each behavioural trait (ranging from 0 to 1), at each observation point, was calculated as the following example:

1 $$\:Valve=\:\frac{\sum\:n\:observations}{n\:Total\:individuals}\:$$

where *n* is the number of individuals in each aquarium (5 in each case) (Verdelhos et al. 2015).

An ‘activity index’ was calculated as the sum of observed traits (ranging from 0 to 5) at each observation point (Verdelhos et al. 2015):

2 $$\ \begin{aligned} \:Activity\:index & = valve + mantle + siphon \\ & + foot + filtration \\ \end{aligned} $$

### Data treatment and statistical analysis

The amplitude of all recorded EOsG responses was measured in millivolts, blank-subtracted, and normalised to the amplitude evoked by the standard stimulus, 10^− 3^ M L-cysteine (similarly blank-subtracted). For each amino acid, thresholds of detection were calculated by linear regression of the concentration-response curves of the data log-transformed. Considering the formula log (*N* + 1.5) = *a*.log *C* + *b*, where *N* is the normalised response, *C* is the concentration, and *a* and *b* are constants, the threshold of detection is the value for *x* where *y* = 0.1761 (i.e. log 1.5; *N* = 0). Thresholds of detection of stimuli “Open and alive” and “Chopped clam” were calculated by linear regression of the dilution-response curves of the data semi log-transformed and estimating the intercept with the *x* axis. Two-way Repeated Measures ANOVA followed by Tukey’s *post hoc* test was applied to assess statistical differences among water conditioned by conspecifics or heterospecific bivalves and their physiological condition (i.e., live but closed, live and open, or chopped dead flesh).

For behavioural data, One-way repeated measures ANOVA or Friedman repeated measures ANOVA on ranks (depending on validation of normality and homogeneity of variances assumptions) was performed to assess for differences throughout the observational points within each stimulus. When applicable, Dunnett’s *post hoc* test was applied. One-way ANOVA or Kruskal-Wallis ANOVA on ranks followed by Holm-Sidak *post hoc* test were applied to compare each stimulus at T0 and T150.

As the present study aimed at a global vision and not an investigation of individual variability, standard error of the mean was chosen as the measure of data dispersion; results are expressed as mean ± standard error of the mean (SEM). The level of significance was set as *P* ≤ 0.05. Statistical analysis was performed using software Sigmaplot (version 15).

## Results

### Sensitivity to amino acids

**Fig. 2:**
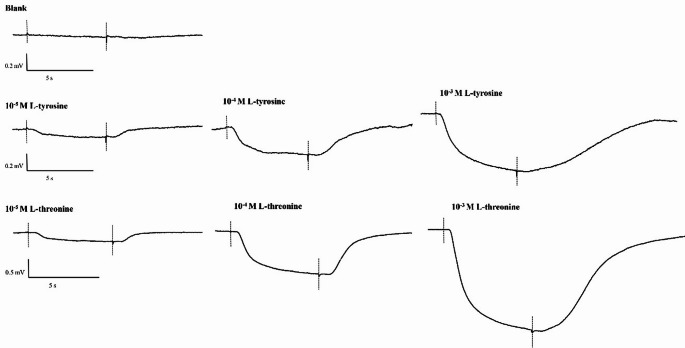
Typical EOsG responses to increasing concentrations (10^− 5^ M – 10^− 3^ M) of L-tyrosine and L-threonine. The dotted lines indicate the beginning and end of stimulus delivery. A downward deflection of the trace is negative

The EOsG responses (Fig. 2) were characterized by a slow and negative deflection at the beginning of the stimulus, followed by a tonic response during which the EOsG showed little or no sign of accommodation. Once the stimulus delivery ended, the potential returned to baseline levels within a few seconds.

Figure 2 shows representative EOsG responses to stimuli, comparing the responses of different concentrations (10^− 5^ M – 10^− 3^ M) of L-tyrosine and L-threonine with the negative control (blank: seawater with no stimulus). L-threonine (10^− 3^ M) evoked stronger responses, with mean amplitudes of 1.12 ± 0.34 mV, followed by L-alanine (1.10 ± 0.30 mV) and L-serine (0.97 ± 0.18 mV). On the other hand, L-tyrosine (10^− 3^ M) evoked responses with lower amplitude (average amplitudes = 0.36 ± 0.08 mV). However, the response with the lowest amplitude was exhibited by L-tryptophan (0.13 ± 0.03 mV).

**Fig. 3:**
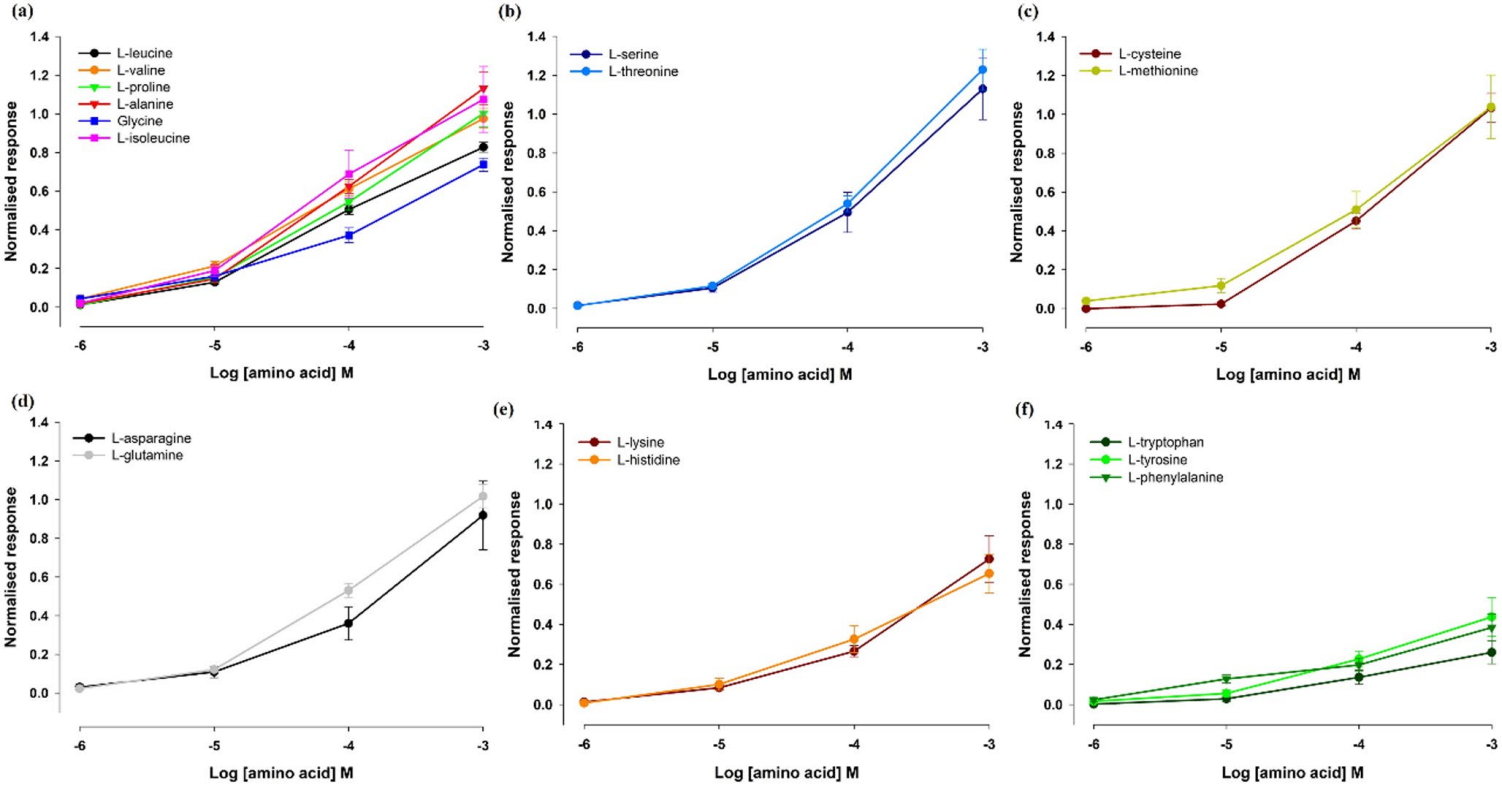
Semilogarithmic plot of the normalised electro-osphradiogram (EOsG) amplitude to amino acids recorded from the osphradium. **a** aliphatic amino acids; **b** hydroxylic amino acids; **c** sulphur-containing aminoacids; **d** amidic amino acids; **e** basic amino acids; **f** aromatic amino acids. Data are shown as mean ± SEM; *N* = 6, except for L-leucine, L-proline and L-cysteine: *N* = 7)

EOsG responses were concentration-dependent, with the different groups of amino acids exhibiting EOsG responses similar in amplitude and shape within each group (Fig. 3). The strongest responses were evoked by hydroxylic amino acids (Fig. 3b), followed by sulphur-containing (Fig. 3c), with normalised responses varying between 1.033 ± 0.08 and 1.23 ± 0.11 for L-cysteine and L-threonine at 10^− 3^ M, respectively. Aromatic amino acids (Fig. 3f) elicited the weakest responses, ranging between 0.26 ± 0.06 and 0.44 ± 0.10 for L-tryptophan and L-tyrosine at 10^− 3^ M, respectively. At higher concentrations (10^− 5^ M – 10^− 3^ M), all groups displayed distinct linear patterns.

**Fig. 4:**
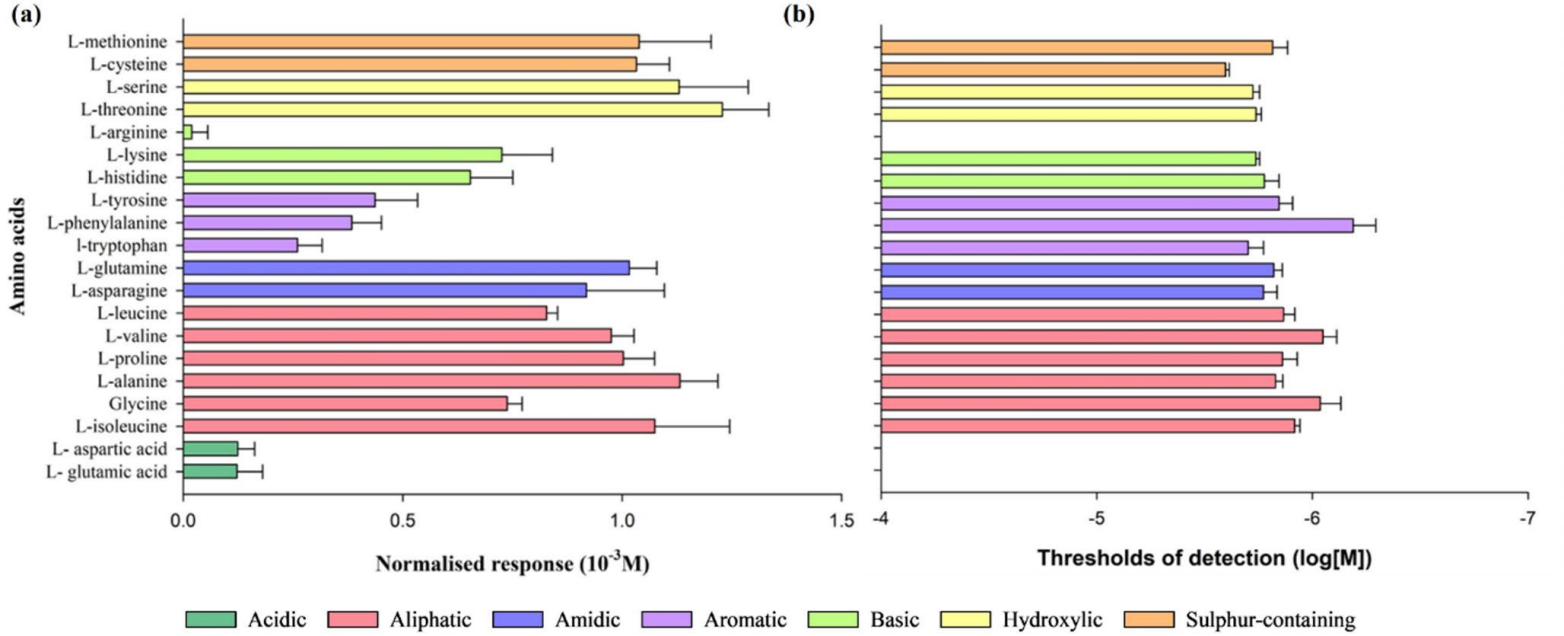
Sensitivity of the osphradium to the twenty proteinogenic amino acids. **a** Normalised responses at 10^− 3^ M and (**b** thresholds of detection of amino acids. Data are shown as mean ± SEM

The thresholds of detection (Fig. 4) varied between 10^− 6.19^ M and 10^− 5.60^ M for L-phenylalanine and L-cysteine, respectively. Nevertheless, the amino acids with the strongest response were not necessarily those with the lowest thresholds (Fig. 4). For example, L-phenylalanine, which evoked a weaker normalised response (0.38 ± 0.07) exhibited the lower threshold of detection (10^− 6.19 ± 0.11^ M), therefore proving to be the most potent amino acid. On the other hand, amino acids that evoked stronger responses, as is the case of L-threonine (1.23 ± 0.11) and L-cysteine (1.03 ± 0.07), showed thresholds of detection of 10^− 5.74 ± 0.02^ M and 10^− 5.60 ± 0.02^ M, respectively. Overall, there was higher variability in amplitude of responses among amino acids than the threshold of detection. As these D.C. voltage responses were recorded in seawater (high conductivity), it is likely that these thresholds are over-estimates (i.e., the true thresholds are lower), as shown in marine fish (Hubbard et al. 2011).

Nevertheless, some amino acids failed to evoke a response, even at the highest concentration tested (10^− 3^ M): the basic amino acid L-arginine and acidic amino acids (L-aspartic acid and L-glutamic acid) (data not shown). Similarly, bile acids (TCA, TLC and cholic acid) (10^− 5^ M) and serotonin (10^− 3^ M) also failed to elicit any response (data not shown).

### Predator-released cues and signals from conspecific and heterospecific bivalves

**Fig. 5:**
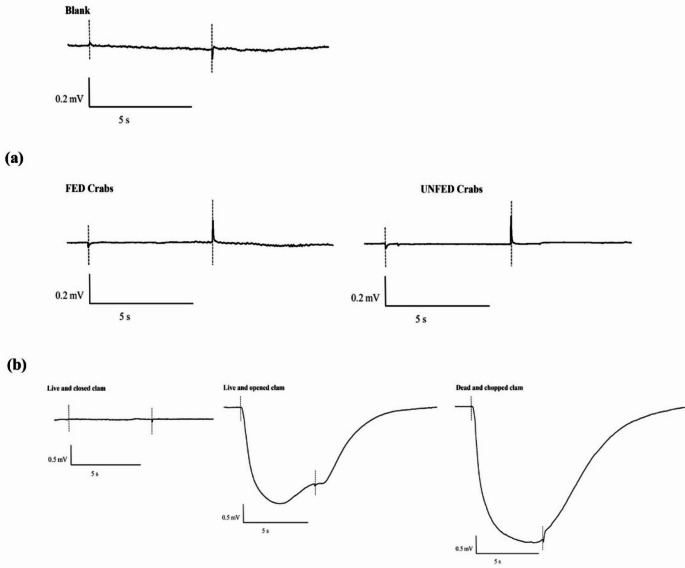
**(a)** EOsG responses to water from fed and unfed crabs in comparison with blank. (**b)** EOsG response to water conditioned with ‘Live and closed’, ‘Live and opened’ and ‘Dead and chopped’ clams, in comparison with blank

**Fig. 6:**
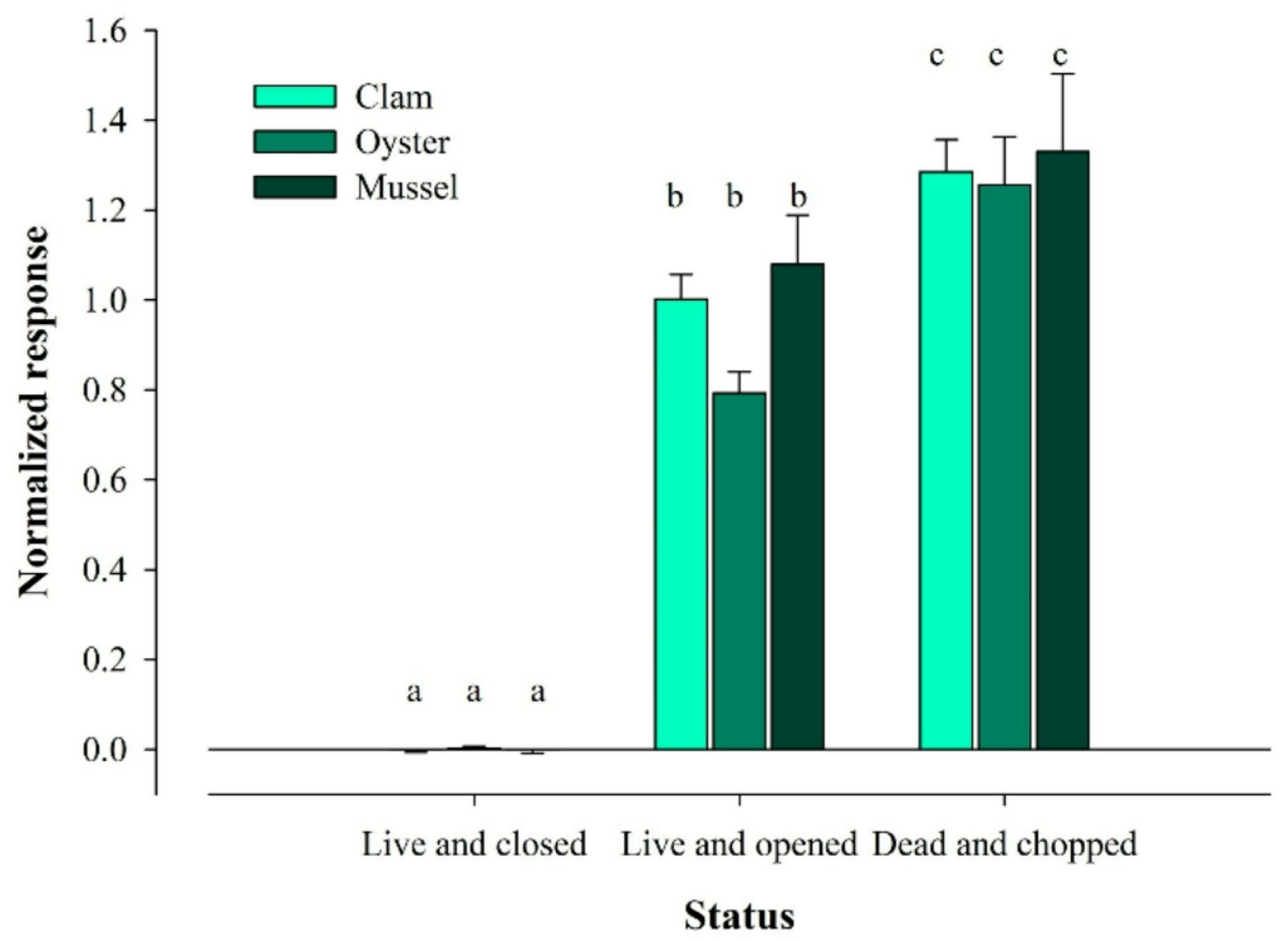
Normalised response to water conditioned with conspecific (clams) and heterospecific bivalves (oysters and mussels), at different conditions (‘Live and closed’, ‘Live and opened’ and ‘Dead and chopped’). Different letters indicate significant differences between different groups (*P* < 0.05)

The first approach compared the response of clams to fed and unfed crabs. Figure 5a illustrates the EOsG response to water from fed and unfed crabs; both failed to elicit a response significantly different from zero (-0.002 ± 0.003 and − 0.001 ± 0.004, respectively). Nevertheless, water conditioned with conspecific and heterospecific bivalves in the three different conditions evoked distinct responses (Fig. 5b). Water conditioned with live and closed bivalves (both conspecific and heterospecific) failed to evoke a response above zero (clam: -0.005 ± 0.006; oyster: 0.002 ± 0.006; mussel: -0.002 ± 0.007) (Fig. 6). However, once one valve was removed, the response increased drastically, varying from 0.79 ± 0.05 to 1.079 ± 0.11, for oyster and mussel-conditioned water, respectively (Fig. 6). If the conspecific and heterospecific bivalves were dead and chopped, the response was slightly higher, evoking responses with amplitudes varying from 1.47 ± 0.78 mV to 1.70 ± 1.05 mV, which corresponded to normalised responses of 1.33 ± 0.17 and 1.29 ± 0.07, in the case of water conditioned with mussels and clams, respectively. Significant differences were observed among the condition of the bivalves (TW RM ANOVA, *F* = 230.998, *d.f.*=2, *p <* 0.001). In contrast, no significant differences were found among species (TW RM ANOVA, *F* = 4.142, *d.f.*=2, *p =* 0.106) nor in the interaction between condition x species (TW RM ANOVA, *F* = 0.626, *d.f.*=4, *p =* 0.657).

### Behavioural analysis

**Fig. 7:**
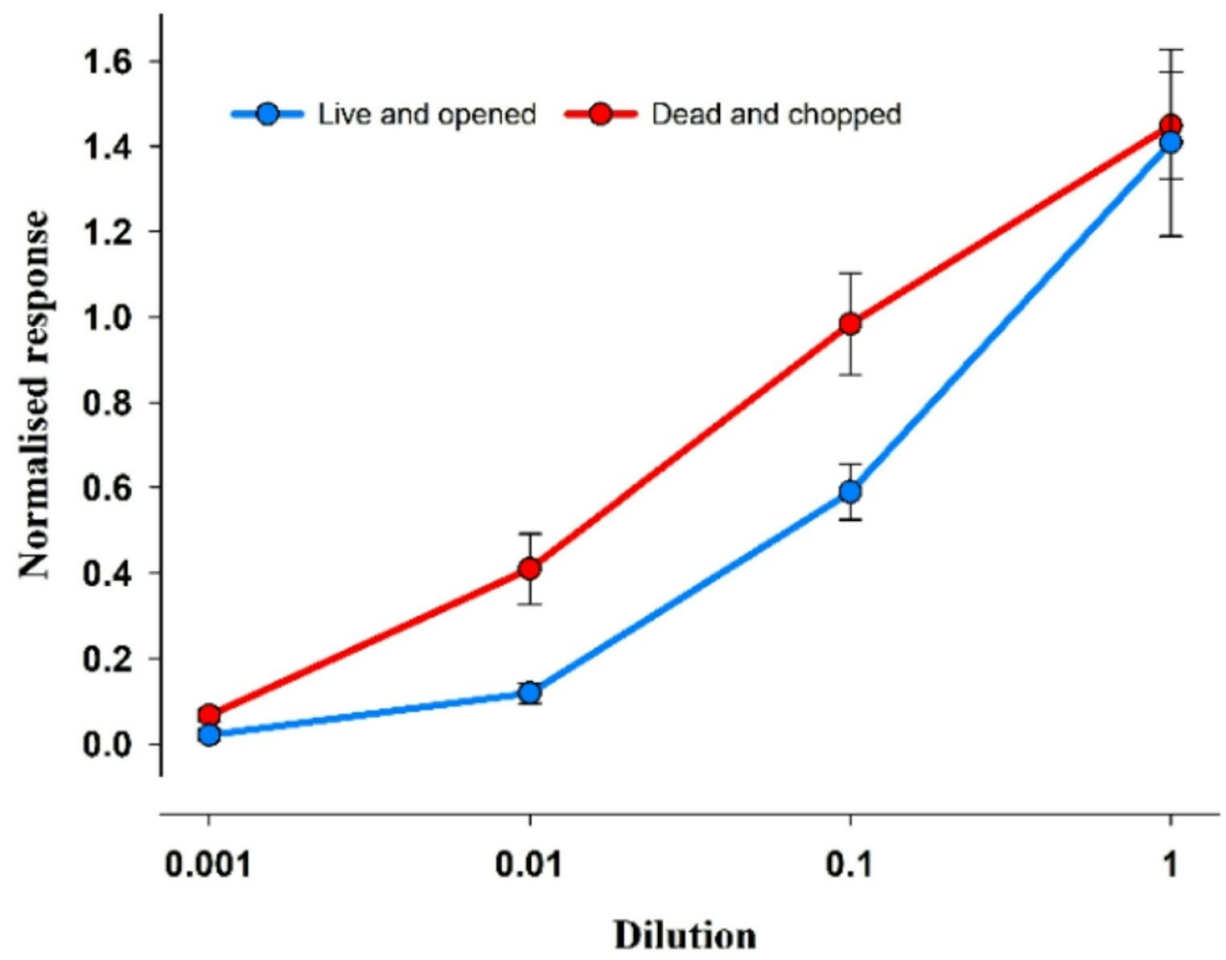
Semilogarithmic plot of the normalised electro-osphradiogram (EOsG) response to stimuli ‘Live and opened’ and ‘Dead and chopped’. Data are shown as mean ± SEM; *N* = 6

Prior to behavioural analysis, it was necessary to determine the EOsG response to a dilution series (Fig. 7) of the stimuli to be used, namely ‘Live and opened’ and ‘Dead and chopped’ – prepared as mentioned above. The normalised response varied between 0.021 ± 0.015 and 1.41 ± 0.22 and 0.067 ± 0.015 and 1.45 ± 0.125 for ‘Live and opened’ and ‘Dead and chopped’, respectively. The amplitude of responses to both stimuli was dilution-dependent, with the stimulus ‘Chopped and dead’ evoking the strongest response, specifically at dilutions of 0.01 and 0.1 (1:100 and 1:10, respectively). The thresholds of detection varied between 10^− 2.10±0.03^ and 10^− 2.86±0.20^ for ‘Live and opened’ and ‘Dead and chopped’, respectively.

**Fig. 8:**
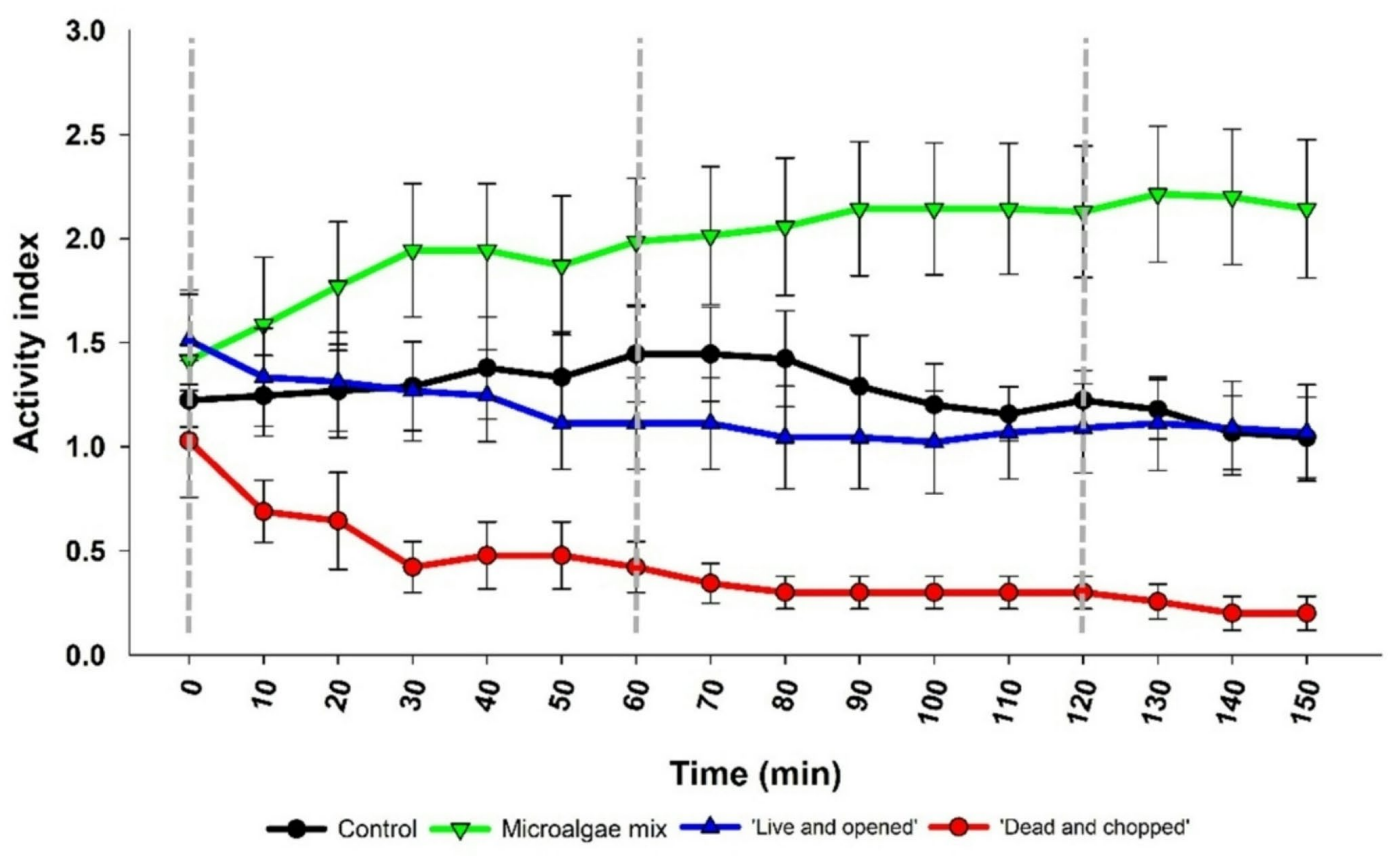
Activity index of clams exposed to different stimuli: ‘Control’ (seawater) (*N* = 9); ‘Microalgae mix’ (*N* = 14); ‘Live and opened’ (*N* = 9) and ‘Dead and chopped’ (*N* = 9). Dotted lines indicate the time of stimulus addition. Data are shown as mean ± SEM

The activity index varied throughout the time, depending on the stimulus to which clams were exposed to (Fig. 8). In ‘Control’, no significant variations were detected from the beginning of the assay (T0; 1.22 ± 0.19) to the end (T150; 1.04 ± 0.19) (Friedman RM analysis of variance on ranks, χ^2^ = 12.247, *d.f.* =15, *p* = 0.660). A similar behavioural pattern was observed after exposure to ‘Live and opened’ stimulus, i.e., there were no significant alterations in clams’ behaviour after 150 min of exposure (Friedman RM analysis of variance on ranks, χ^2^ = 23.339, *d.f.* =15, *p* = 0.077). However, a slight decrease in activity index was observed, varying between 1.51 ± 0.24 (T0) to 1.07 ± 0.23 (T150). After the addition of a positive stimulus, such as ‘Microalgae mix’, clam activity significantly increased, ranging from 1.41 ± 0.32 at T0 to 2.14 ± 0.33 at T150 (One-way RM ANOVA, *F =* 7.206, *d.f.* = 15, *p* < 0.001). Conversely, a significant decrease was observed after exposure to ‘Dead and chopped’ stimulus, from 1.03 ± 0.27 at T0 to 0.20 ± 0.08 at T150 (Friedman RM analysis of variance on ranks, χ^2^ = 67.181, *d.f.* =15, *p* < 0.001). As expected, at T0 no significant differences were observed among stimuli (KW, *H* = 2.132, *d.f.* = 3, *p* = 0.545). At the end of the trial, the activity index under the ‘Microalgae mix’ stimulus was significantly different from ‘Control’ (One-way ANOVA, *F =* 9.890, *d.f.* = 3, *p* = 0.024), ‘Live and opened’ (One-way ANOVA, *F =* 9.890, *d.f.* = 3, *p* = 0.024) and ‘Dead and chopped’ stimuli (One-way ANOVA, *F =* 9.890, *d.f.* = 3, *p* < 0.001). Notwithstanding, when only considering ‘Control’ and ‘Live and opened’ and ‘Dead and chopped’, it was possible to detect that ‘Dead and chopped’ stimulus was significantly different from ‘Live and opened’ and from ‘Control’ at T150 (One-way ANOVA, *F =* 7.509, *d.f.* = 2, *p* = 0.007).

All behavioural traits (valve, mantle, siphons, filtration and foot) were positively influenced by the stimulus ‘Microalgae mix’ (supplementary data, fig. S2-S6). In general, the presence of the valve (fig. S2) was always associated with the mantle (fig. S3) and, except for the ‘Dead and chopped’ stimulus, the frequency of these traits remained above 0.4 throughout the trial. The frequency in ‘Control’ group remained almost constant from T0 to T150 while decreased in stimuli ‘Live and opened’ and ‘Dead and chopped’. The frequency of siphons (fig. S4) decreased in ‘Live and opened’ and ‘Dead and chopped’ stimuli. Even though the presence of siphons is generally associated with filtration, in this study, we considered that clams were filtering when the inhalant siphon was completely open (fig. S1), and it was possible to observe water movement. Thus, filtration (fig. S5) only occurred after exposure to ‘Microalgae mix’, increasing throughout the trial (0.23 ± 0.08 to 0.45 ± 0.08), while decreasing close to 0 in the ‘Control’ stimulus (0.09 ± 0.06 to 0.02 ± 0.02). No filtration was observed in calms exposed to ‘Live and opened’ and ‘Dead and chopped’ stimuli. Similarly, foot (fig. S6) was absent in ‘Live and opened’ and ‘Dead and chopped’ stimuli during the observation period and the maximum frequency observed was of 0.13 ± 0.03 for clams exposed to ‘Microalgae mix’.

## Discussion

The clam osphradium proved to be sensitive to most L-amino acids. The form of electrophysiological response (EOsG) from the osphradium in clams was similar to that of Pacific oysters (Rato et al. 2023) and Mediterranean mussels (Cozzolino et al. 2024). It is characterized by a slow negative deflection, followed by a prolonged tonic phase, which returns to baseline levels within seconds once the stimulus delivery ends. Contrary to fishes, the EOsG response in bivalves is of lower amplitude and lacks the initial fast-adapting phasic response (e.g., Modesto et al. 2024).

When comparing the responses of clams and oysters to amino acids, the amplitude of the normalised response evoked by 10^− 3^ M L-cysteine in clams (0.26 ± 0.05 mV) was about half of that evoked by the same amino acid in oysters (0.53 ± 0.09 mV); in turn, 10^− 3^ M L-serine elicited a slightly higher amplitude in clams (0.97 ± 0.18 mV) than oysters (0.85 ± 0.21 mV) (Rato et al. 2023). Clams showed normalised responses with wider variability among amino acids than those of oysters, and with larger differences among different odorants. In clams, the most potent amino acid was L-threonine, and the least potent of those that evoked any response was L-tryptophan, whereas in oysters, the most potent amino acid was L-asparagine while the least potent one detected was L-tyrosine (Rato et al. 2023). Nevertheless, the sensitivity of clams to amino acids appears to be slightly lower than that of oysters, with thresholds of detection varying between 10^− 6.19^ M and 10^− 5.60^ M. Moreover, the amino acids with lower thresholds were not necessarily those evoking higher EOsG amplitudes, as previously described in the Mozambique tilapia (*Oreochromis mossambicus*) (Kutsyna et al. 2016) and in the oyster *Magallana gigas* (Rato et al. 2023). For example, L-phenylalanine had the lowest threshold of detection, but evoked lower amplitude EOsGs, whereas L-threonine elicited a strong response, with larger amplitude, and yet the threshold of detection was higher. These differences may result from adaptation to different habitats. Although both species inhabit intertidal areas, clams live in close association with the substrate, spending their benthic life buried. In contrast, oysters usually settle on structures in the open water column and, living in the intertidal zone, are exposed to the air for several hours a day. Moreover, food availability and microalgae composition may also vary between the water column to substrate, which may explain the slightly different sensitivities to amino acids. Clams were more sensitive to hydroxylic amino acids (e.g. L-threonine and L-serine) and less sensitive to aromatic amino acids (e.g. L-tryptophan and L-phenylalanine). That acidic amino acids (L-aspartic acid and L-glutamic acid) failed to evoke a response is consistent with previous studies, wherein acidic amino acids evoke small responses in marine fishes and none in oysters (Velez et al. 2005; Hubbard et al. 2011; Rato et al. 2023). Although amino acids are potent odorants across several aquatic taxa, and are generally thought to be involved in feeding or food search (e.g. Arzt et al. 1986; Bailey and Laverack 1966; Fuzessery and Childress 1975; Hara 2006; Heerema et al. 2018; Magel et al. 2007), this has yet to be tested experimentally in bivalves.

The perception of predation risk and, consequently, the survival of prey species are strongly dependent on their ability to detect alarm cues or predator-released kairomones and respond accordingly (Johnson and Smee 2012). In the current study, predator-released cues, from either fed or unfed crabs, failed to evoke an electrophysiological response in the osphradium. This is consistent with Hubert et al. (2023); mussels did not respond behaviourally to chemical cues released by crabs when caged, but closed their valves when in physical contact with free-moving crabs. Similarly, Griffiths and Richardson (2006) observed that *Macoma balthica* did not respond to cues released from starved crabs, failing to increase burial depth. However, when exposed to cues from crabs feeding on conspecifics or heterospecifics (cockle), *M. balthica* burial depth increased. Bivalves therefore may use other cues to detect potential predators; anecdotal evidence suggests the same with blue crabs and oysters (Carroll and Clements 2019). Thus, the chemical cues are likely released by the injured prey rather than the predator; water conditioned by conspecific and heterospecific bivalves, simulating injured and stressed prey (live with one valve removed, and consumed prey (dead with chopped flesh) evoked a strong electrophysiological response in the osphradium.

Some previous studies (e.g., Flynn and Smee 2010Smee and Weissburg 2006b) were performed in the field with caged predators; clams increased their burial depth and decreased feeding in the presence of nearby caged predators feeding on conspecifics (Smee and Weissburg 2006b; Flynn and Smee 2010). This suggests that clams detect cues released by conspecifics, rather than those released by predators, since the remains of consumed prey are still present in the water. Similarly, effluent from crabs crushing and feeding on con- and heterospecifics (*Cerastoderma edule*) elicited a strong behavioural response in *M. balthica*; a sharp increase in burial depth (Griffiths and Richardson 2006). These authors suggested that the breakdown products of the crab diet as well as cues from breakdown products from related species are the signals that *M. balthica* responds strongly to. In the current study, the activity index of clams decreased after exposure to water conditioned with injured or dead conspecifics – ‘Live and opened’ and ‘Dead and chopped’, respectively – with the latter stimulus inducing a sharp decrease. Although both stimuli evoked strong responses from the osphradium, the behavioural response to dead and chopped conspecifics was much stronger. This suggests that severe tissue damage releases more and/or different odorants than simply opening one valve, and that these additional odorants are necessary to evoke the appropriate anti-predator behaviour. It is possible, however, that the odorant(s) released by live and opened clams – possibly *via* the haemolymph – serve as a ‘warning’ to alert them to possible danger. As such alarm cues are likely complex mixtures; we believe that the EOsG will prove a valuable tool to isolate and identify the individual components of such mixtures, given that each component alone probably will not evoke the full behavioural response.

That clams did not detect chemical cues from crabs, regardless the condition (fed or unfed), whereas cues released by injured prey evoked strong electrophysiological responses, may be due to the energetic cost associated with the exhibition of predator avoidance behaviours (Scherer et al. 2016). Clams should be able to distinguish between possible and real predation threats, in order to provide suitable defences (Cronin 2001). Predator avoidance involves an energetic investment in defensive behaviours and structures, which may result in reduced feeding and negatively affect growth and reproduction (De Meester et al. 1999; Preisser et al. 2005; Kobak and Ryńska 2014). Considering this, it is not energetically beneficial for clams to always react to potential predators, since they may not pose a real threat.

According to Büchner-Miranda et al. (2024), however, the magnitude of prey responses to predator-released cues may be dependent on the signal intensity; i.e., different densities of predators may indicate distinct predation risks to which prey will respond appropriately (Von Elert and Pohnert 2000; Büchner-Miranda et al. 2024). Moreover, predator-released cues may not be recognized innately (Chivers et al. 1996). Considering this, the intensity of the signal to which clams were exposed to may not have been strong enough to elicit an electrophysiological response, and consequently to be perceived as a potential predatory threat.

The current study also suggests some overlap among bivalve alarm cues; clams responded to similar cues from both oysters and mussels. Whether the behavioural responses are similar remains to be tested. This ability to detect cues from both injured conspecifics and closely-related species (Schoeppner and Relyea 2005), may compensate for their failure to detect predators (Smee and Weissburg 2006a). Living in close proximity to conspecifics, in dense aggregation beds, may be advantageous for prey organisms that are able to detect chemical cues from injured prey but not the predators themselves, since consumption of neighbours represents an immediate and clear predatory threat (Mathis and Smith 1993; Smee and Weissburg 2006a).

Furthermore, clams may detect potential predatory threats through a combination of chemical and mechanical cues. Flynn and Smee (2010) observed that *M. arenaria* burial depth increased in the presence of green crabs consuming conspecifics and after artificial tactile stimulation, therefore suggesting that both chemical and tactile cues are crucial for sensing potential predatory threats, in which chemical signals act as an initial warning, whereas tactile signals pose an immediate threat. Likewise, the gaping in *Crassostrea virginica* did not cease in the presence of blue crabs until they came into physical contact with them (Carroll and Clements 2019).

It seems that alarm cues in bivalves share common characteristics with those of fish from the original observation of Karl von Frisch (von Frisch 1942) in that they both have innate anti-predator responses to alarm cues released by damaged conspecifics (Ferrari et al. 2010; Maximino et al. 2019). However, in the case of fishes, there is the capacity to learn by association with other stimuli. Whether this is the case in bivalves awaits further investigation.

## Conclusion

In the natural environment, chemical cues from injured prey represent the first signal of alarm which, combined with tactile stimulation from a foraging predator, indicates a real predatory event. However, it would be energetically costly for clams to always react to predators when they do not pose a real risk. Further research is needed to understand if and how clams make use of odorants released by injured heterospecifics at a behavioural level; are the chemical similar to those released by conspecifics? Clearly, it would also be important to isolate and identify the chemicals involved. We suggest that the EOsG will be a valuable tool in such work. Furthermore, understanding how bivalves detect and respond to not only to alarm cues but chemical signals in general sheds light on their ecological interactions and offers insights that may be relevant for aquaculture management.

## Supplementary Information

Below is the link to the electronic supplementary material.

Supplementary Material 1

## Data Availability

No datasets were generated or analysed during the current study.
