# Supplementary material for: Electrophysiological responses of the clam (Ruditapes decussatus) osphradium to amino acids and alarm cues
Source: J Comp Physiol A Neuroethol Sens Neural Behav Physiol. 2025 Sep 5;211(5-6):561–73. doi: 10.1007/s00359-025-01757-2 (PMC12592294; doi:10.1007/s00359-025-01757-2)
Supplement: Supplementary file 1 — Supplementary Material 1 [file 359_2025_1757_MOESM1_ESM.docx]

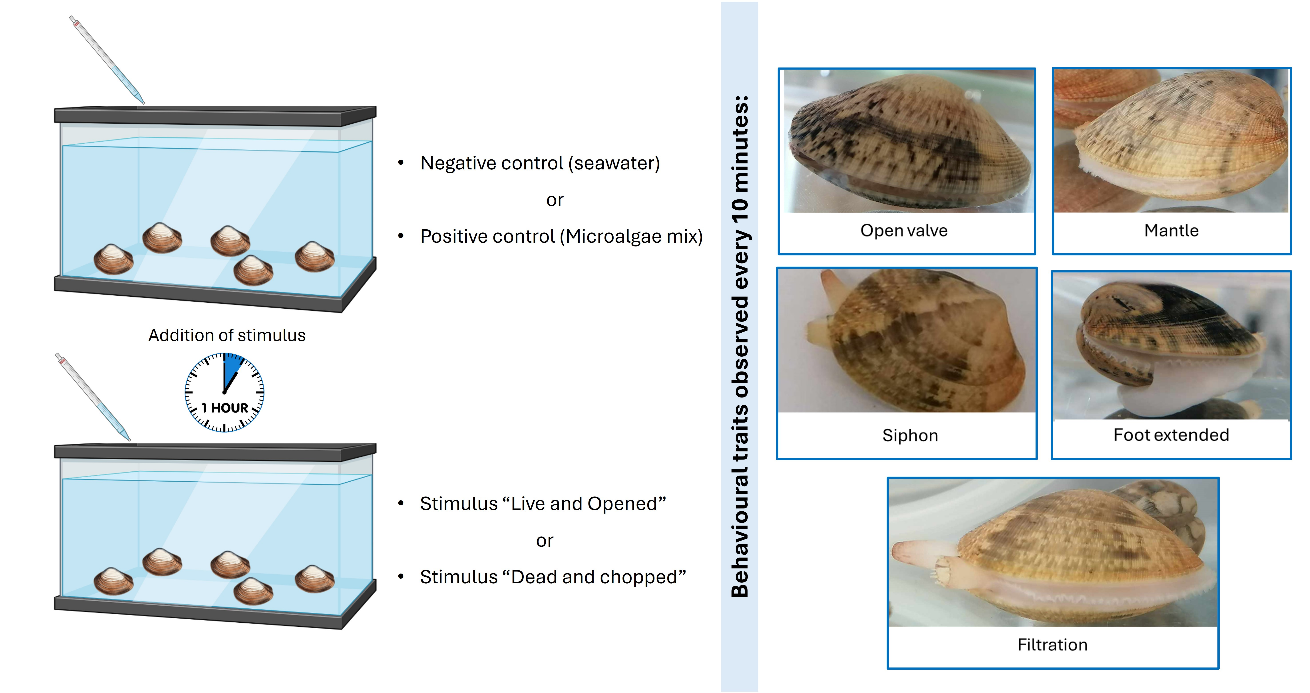
**Fig. S1** Experimental set-up of behavioural assays. Five clams (*R. decussatus*) per aquarium were exposed to different stimuli – seawater (‘Control’) and ‘Microalgae mix’ (used as negative and positive control, respectively) and water conditioned with injured conspecifics (‘Live and opened’) and dead and chopped conspecifics (‘Dead and chopped’). Stimuli were added every hour. Behavioural traits (‘open valve’; ‘mantle’; ‘siphons; ‘foot’ and ‘filtration’) were observed every 10 minutes


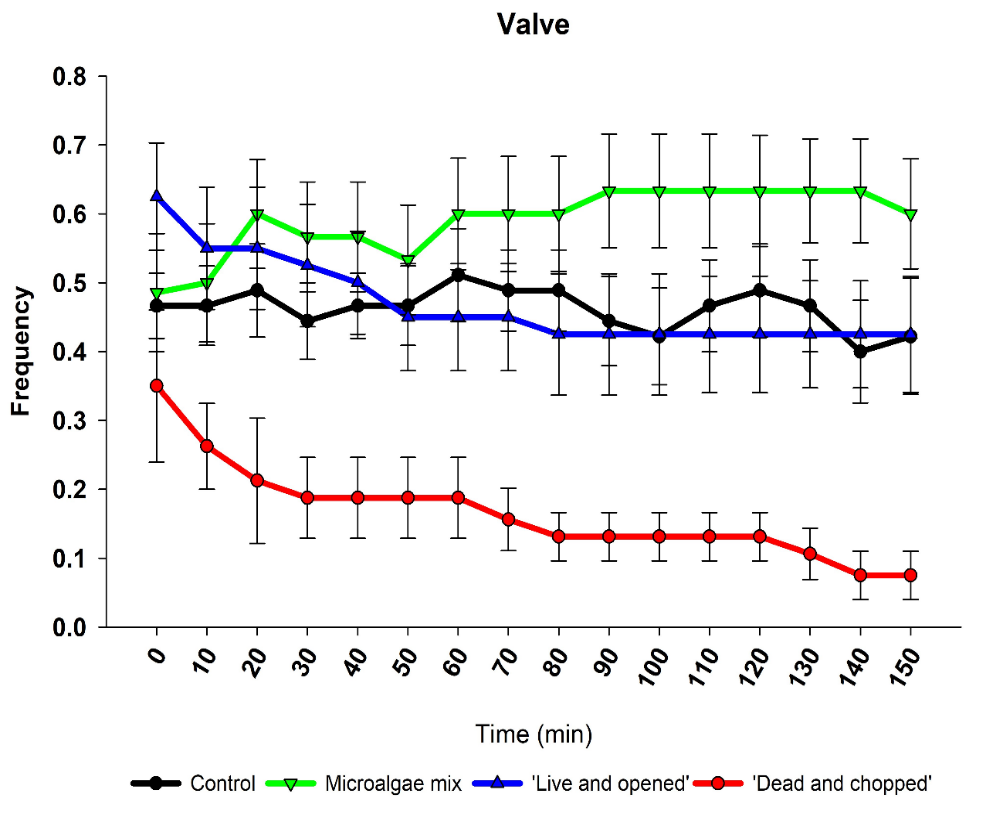


**Fig. S2** Frequency of the behavioural trait ‘Valve’ throughout the observation period, after exposure to different stimuli (‘Control’, ‘Microalgae mix’, ‘Live and opened’ and ‘Dead and chopped’). Dotted lines indicate the time of stimulus addition


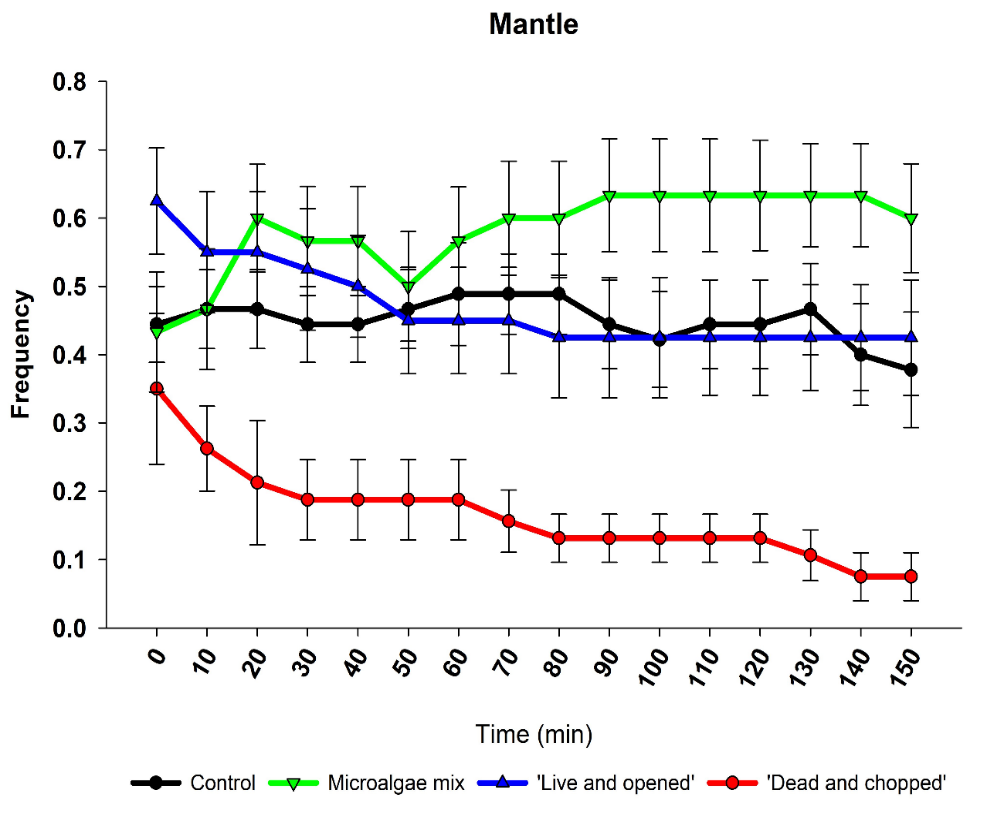


**Fig. S3** Frequency of the behavioural trait ‘Mantle’ throughout the observation period, after exposure to different stimuli (‘Control’, ‘Microalgae mix’, ‘Live and opened’ and ‘Dead and chopped’). Dotted lines indicate the time of stimulus addition


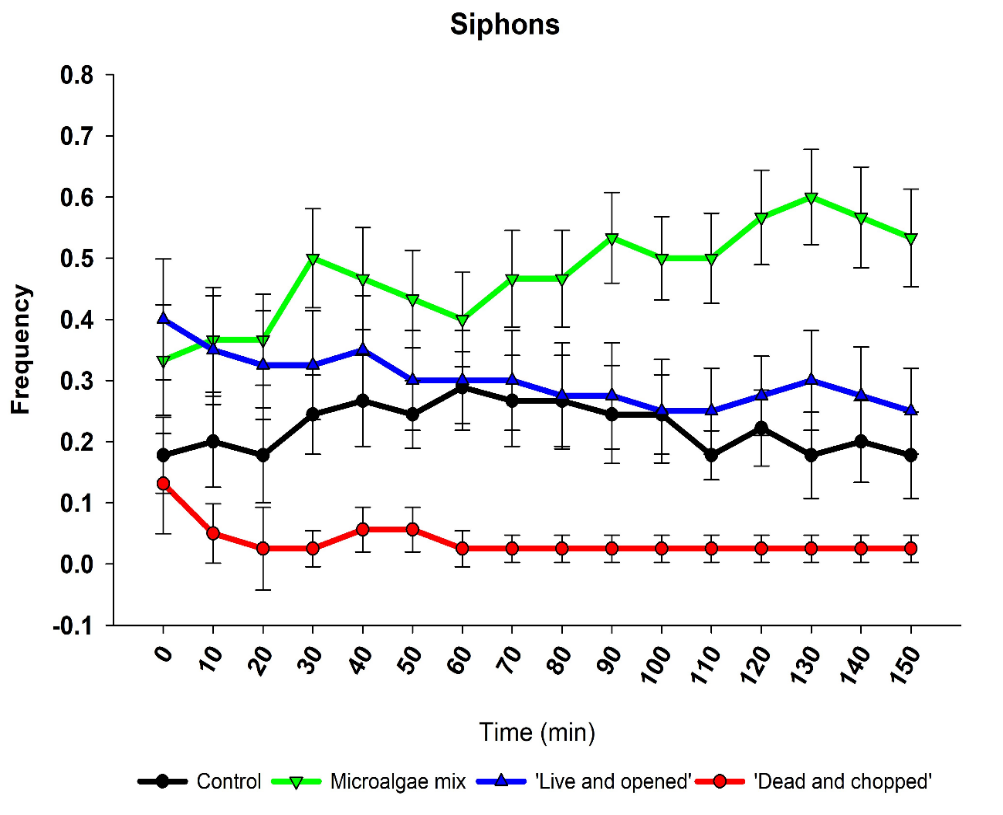


**Fig. S4** Frequency of the behavioural trait ‘Siphons’ throughout the observation period, after exposure to different stimuli (‘Control’, ‘Microalgae mix’, ‘Live and opened’ and ‘Dead and chopped’). Dotted lines indicate the time of stimulus addition


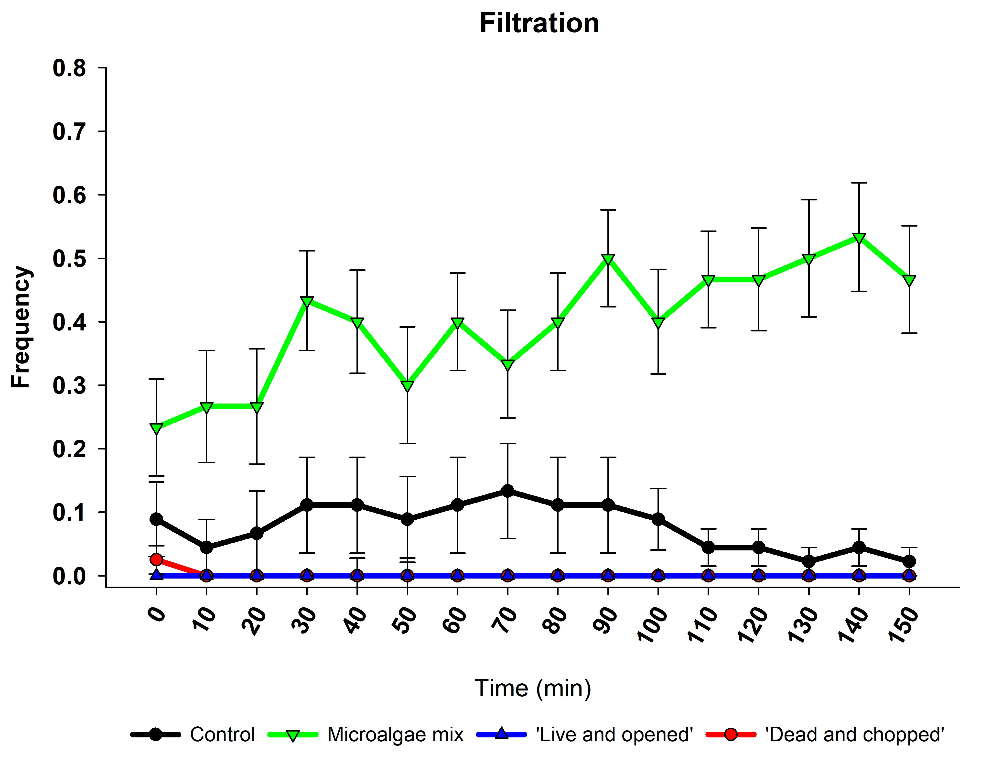


**Fig. S5** Frequency of the behavioural trait ‘Filtration’ throughout the observation period, after exposure to different stimuli (‘Control’, ‘Microalgae mix’, ‘Live and opened’ and ‘Dead and chopped’). Dotted lines indicate the time of stimulus addition


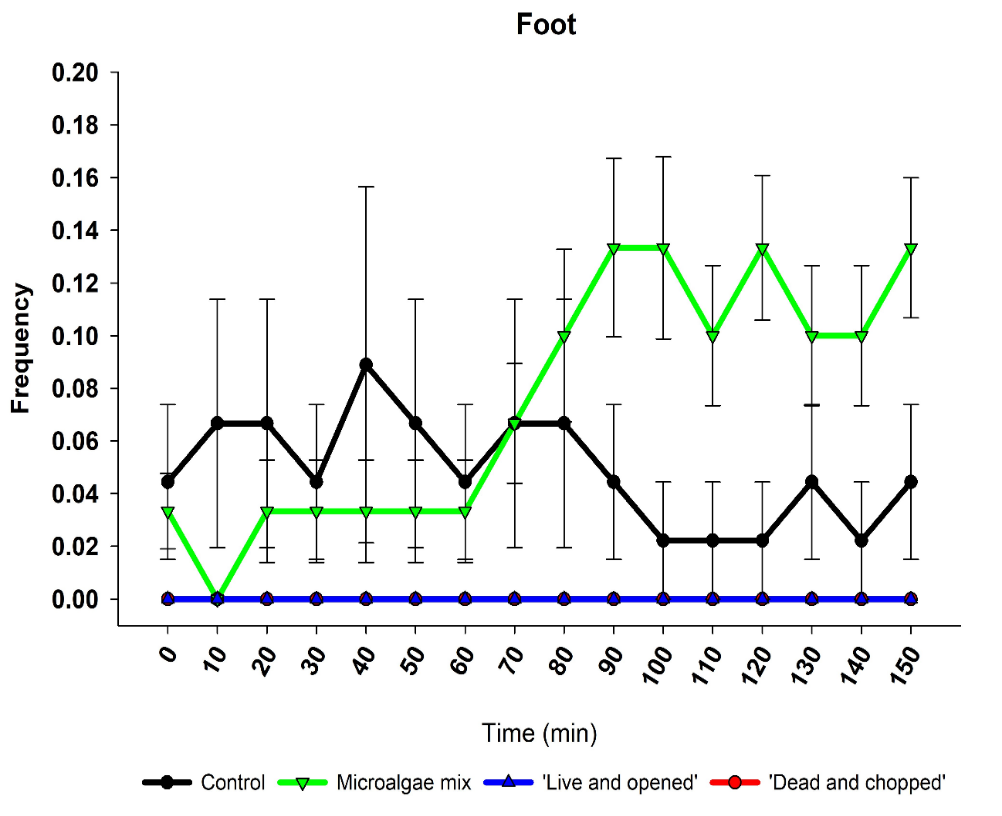


**Fig. S6** Frequency of the behavioural trait ‘Foot’ throughout the observation period, after exposure to different stimuli (‘Control’, ‘Microalgae mix’, ‘Live and opened’ and ‘Dead and chopped’). Dotted lines indicate the time of stimulus addition
